# The Influence of Laser Surface Remelting on the Tribological Behavior of the ECAP-Processed AZ61 Mg Alloy and AZ61–Al_2_O_3_ Metal Matrix Composite

**DOI:** 10.3390/ma13122688

**Published:** 2020-06-12

**Authors:** Beáta Ballóková, Ladislav Falat, Viktor Puchý, Zuzana Molčanová, Michal Besterci, Róbert Džunda, Aqeel Abbas, Song-Jeng Huang

**Affiliations:** 1 Institute of Materials Research, SAS, Watsonova 47, 04001 Košice, Slovakia; bballokova@saske.sk (B.B.); vpuchy@saske.sk (V.P.); molcanova@saske.sk (Z.M.); mbesterci@saske.sk (M.B.); rdzunda@saske.sk (R.D.); 2 Department of Mechanical Engineering, National Taiwan University of Science and Technology, No. 43, Section 4, Keelung Road, Taipei 10607, Taiwan; engr.aqeel14@gmail.com

**Keywords:** AZ61 magnesium alloy, AZ61–Al_2_O_3_ composite, laser surface remelting, coefficient of friction, wear rate

## Abstract

This paper deals with the tribological study of the laser remelted surfaces of the ECAP-processed AZ61 magnesium alloy and AZ61–Al_2_O_3_ metal matrix composite with 10 wt.% addition of Al_2_O_3_ nanoparticles. The study included the experimental optimization of the laser surface remelting conditions for the investigated materials by employing a 400 W continual wave fiber laser source. Tribological tests were performed in a conventional “ball-on-disc” configuration with a ceramic ZrO_2_ ball under a 5 N normal load and a sliding speed of 100 mm/s. The results showed that both the incorporation of Al_2_O_3_ nanoparticles and the applied laser treatments led to recognizable improvements in the tribological properties of the studied AZ61–Al_2_O_3_ composites in comparison with the reference AZ61 alloy. Thus, the best improvement has been obtained for the laser modified AZ61–10 wt.% Al_2_O_3_ nanocomposite showing about a 48% decrease in the specific wear rate compared to the laser untreated AZ61 base material.

## 1. Introduction

Particle-reinforced magnesium matrix composites have already been used for the production of structural components in many industrial applications (e.g., in aerospace, automotive, nuclear, and biomedical applications, etc.) because of their high strength-to-weight ratio and good formability [1,2,3,4,5,6]. The properties of many alloys, in terms of their strength and hardness, may be enhanced by the presence of small, uniformly dispersed nanoparticles, such as the SiC or Al_2_O_3_ ceramic nanoparticles, carbon nanotubes (CNTs), and others, within the original metal matrix, in order to form the metal matrix composites (MMCs). The main challenges for the processing of MMCs are related to obtaining a satisfactorily homogeneous dispersion of the reinforcing particles in the metal matrix, the formation of strong interfacial bonding, and also the chemical and structural stability of the dispersed particles [7,8,9]. These dispersoids can be introduced into the composite as insoluble particles during the powder compaction (dispersion strengthening) or they can be formed as precipitates via in-situ solid state reactions (precipitation or age hardening). Such incorporated particles may effectively block the dislocation motion in metal matrix composite materials, leading to their increased strength, stiffness, and hardness [10]. Besides dispersion strengthening, a convenient way for obtaining desirable mechanical properties (i.e., higher strength and hardness) is related to the creation of very fine, submicron-grained microstructures [11], utilizing the Hall–Petch relationship. Severe plastic deformation (SPD) techniques represent metal-forming processes inducing complex stress states and high shear strains within the processed materials, with the resulting microstructures showing high-density defects, ultra-fine grain sizes, and nano-crystalline structures [12,13]. For instance, equal channel angular pressing (ECAP), accumulative roll bonding (ARB), high-pressure torsion (HPT), mechanical alloying (MA), and asymmetric rolling (ASR) represent some typical routes used in manufacturing industries so far, besides other SPD techniques. The effects of variation of the ECAP-processing conditions on the mechanical properties and microstructure of magnesium alloy were extensively studied, e.g., in [14,15,16,17].

Apart from SPD processing, surface modification techniques, e.g., by laser treatment, represent further possibilities for obtaining desirable material properties. Since wear and corrosion are essentially surface-related degradation processes, they can, in principle, be reduced by appropriate tailoring of the surface microstructure. A high-power laser beam may be used as a source of heat to melt the near-surface region of a substrate in order to improve the surface dependent properties [18]. Since the melting and solidification processes occur within a very short interaction time and remain confined only to the top surface of the laser treated material, the bulk underneath acts as an infinite heat sink without any noticeable change in microstructure. The large temperature gradient across the boundary between the melted surface and underlying substrate results in rapid self-quenching and re-solidification [19]. Laser surface modification was reported to improve the wear resistance of various engineering materials including conventional tool steels and light-metal alloys [20,21]. Laser surface alloying of the AZ91E alloy with incorporated WC and TiC particles resulted in the significant enhancement of the alloy’s sliding wear resistance [22].

Our previous works [23,24,25] dealt with investigations of various AZ61 MMCs in terms of their detailed microstructural analyses, the optimization of superplasticity parameters, and the evaluation of deformation and fracture behavior by means of in-situ tensile tests in a scanning electron microscope (SEM). The microstructural analyses included the evaluation of matrix grain size, the average size of the incorporated dispersoids (SiC or Al_2_O_3_), and their distribution. It has been revealed that the microstructures of experimental materials exhibited average matrix grain sizes of about 8 μm. Moreover, there was a slight tendency towards smaller grain sizes in materials with 10 wt.% of Al_2_O_3_ particles. It was also reported [25,26,27,28] that the hot deformation process of such materials leads to the creation of coherent Mg_17_Al_12_ intermetallic precipitates. 

The aim of the present work was to investigate and discuss potential improvements in the tribological behavior of the laser modified surface layers of the ECAP-processed AZ61 alloy and AZ61–Al_2_O_3_ metal matrix composite with 10 wt.% addition of Al_2_O_3_ nanoparticles by continual wave fiber laser treatment, employing optimized laser processing parameters.

## 2. Materials and Methods 

The original metal matrix material used in the present work was commercial magnesium alloy AZ61 produced by Metaltech Industrial Co, LTD, Taiwan. Its chemical composition is shown in Table 1.

The particles of Al_2_O_3_ with a weight fraction of 10% were used as the reinforcement phase. The commercially available Al_2_O_3_ powder with an average particle diameter of about 20 nm and a purity of 99.8% was added into the AZ61 alloy melt to form Mg-based metal-matrix composites. The melt-stirring technique was used to fabricate the AZ61–Al_2_O_3_ magnesium alloy matrix composite. Detailed information on the melting procedure that was used is published in [29]. 

**Figure 1:**
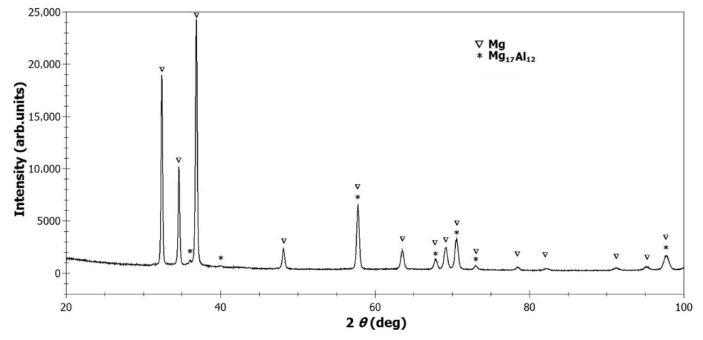
XRD pattern of AZ61–10 wt.% Al_2_O_3_ nanocomposite indicating the occurrence of Mg matrix and Mg_17_Al_12_ intermetallic phase.

The cast AZ61–Al_2_O_3_ composite as well as the AZ61 alloy were further processed by ECAP which was carried out in a die with a die angle of 120°. The deformation temperature was 573 ± 10 K. During deformation, the plunger speed was about 1.0 mm/s. After each extrusion pass, the billet was quenched in water. The billets were rotated counter-clockwise around the exit extrusion axis by 90° between each pass, the so-called Bc route, and each bar was ECAP-processed by 4 passes. The ECAP-processed materials were subjected to X-ray diffraction (XRD) phase analysis which was carried out on a Philips X’Pert Pro diffractometer (PANalytical B.V., Almelo, The Netherlands) in Bragg–Brentano geometry, using Cu-Kα radiation and the ultra-high-speed detector X’Celerator (type number: 9430 030 15201, Malvern Panalytical Ltd., Malvern, UK). The obtained XRD pattern from the performed XRD measurement on the AZ61–10 wt.% Al_2_O_3_ composite is shown in Figure 1. It shows the presence of the Mg matrix and the Mg_17_Al_12_ phase. The occurrence of the Al_2_O_3_ phase was not detected due to its nanometric size. The visualization of Al_2_O_3_ dispersoids by transmission electron microscopy (TEM Tesla BS 500, Tesla Brno, Brno, Czech) was shown in our previous studies [23,27] on AZ61–Al_2_O_3_ composite systems. 

From the ECAP-processed materials, cylindrical specimens (20 mm in diameter and 5 mm in height) were prepared for laser surface treatment. The prepared samples of AZ61 alloy and AZ61–Al_2_O_3_ nanocomposite were mounted in a sample holder and set into the experimental laser workstation TRUMPF 3003 (TRUMPF GmbH + Co. KG, Ditzingen, Germany) with laser source TruFiber400 (TruFiber400 (G2), TRUMPF Laser GmbH + Co. KG, Schramberg, Germany). The samples were laser treated in an air atmosphere using a programmable focused optic head. The basic characteristics of the used laser were as follows: 400 W laser power, 50 kHz modulation frequency, and 1064 nm wavelength of the beam radiation. The optimization of the laser surface treatment conditions was performed via optimizing the laser beam defocusing and laser beam travel speed. The optimization was aimed at obtaining beneficial morphological characteristics of the laser-affected zone (LAZ) without pronounced material ablation. The examined defocusing was in range from +5 to +10 mm and the laser beam travel speed was from 5 to 40 mm/s with respect to the treated sample surface. 

Microstructural analyses of the individual materials and their LAZs were carried out on prepared metallographic cross-sections using a light-optical microscope (LOM) OLYMPUS GX71 (OLYMPUS Europa Holding GmbH, Hamburg, Germany). The cross-sectional samples were mechanically ground on the SiC grinding papers with granularity from 500 to 1000 grit, then they were polished using a diamond paste suspension with a particle size ranging from 1 to 0.25 μm and finally etched in 10% acetic acid. After the completion of the microstructural analyses, the prepared cross-sections were further subjected to nanoindentation measurements using the micro-nano indenter TTX NHT2 (CSM Instruments Inc., Needham, MA, USA). The depth sensing indentation (DSI) technique was used to determine the indentation hardness (H_IT_) and elastic modulus (E_IT_). The indentation measurements were performed linearly on the sample cross-sections. The Berkovich diamond indenter was used in the normal mode, loading at 5 Hz frequency and 5 mN load amplitude. The loads of up to 100 mN were applied. The resulting load-penetration curves were analyzed according to the method of Oliver and Pharr [30] and the values of H_IT_ and E_IT_ were calculated. Up to 20 indentations were performed for each tested material and the obtained data were statistically evaluated.

The tribological behavior of both the AZ61 alloy and the AZ61–Al_2_O_3_ composite in all their material states (i.e., without or with laser surface treatment) was studied in dry sliding conditions using the universal tribometer HTT (CSM Instruments Inc., Needham, MA, USA). The measurements were performed in air at room temperature, using the conventional “ball-on-disc” technique according to the ASTM G 99 – 95a standard [31]. Prior to the tribological testing, the top surfaces of the studied materials (i.e., both the laser untreated materials as well as the laser surface remelted ones) were mechanically ground on dry SiC grinding papers with their granularity ranging from 500 to 1200 grit. The tribological partner for each tested material was a polished ZrO_2_ ball with 6 mm in diameter. The applied load, sliding speed, and sliding distance were 5 N, 100 mm/s, and 50 m, respectively. In order to identify the acting wear mechanisms, the morphological characteristics of the worn surfaces were analyzed using the scanning electron microscope (SEM) Tescan Vega-3 LMU (TESCAN Brno, s.r.o., Czech Republic). Complementary analyses of the local chemical composition within the wear tracks, including the single point analyses as well as elemental mapping analyses, were performed using the energy dispersive X-ray (EDX) spectrometer Bruker XFlash Detector 410-M (Bruker Nano GmbH, Berlin, Germany).

## 3. Results and Discussion

### 3.1. Microstructural Observations

**Figure 2:**
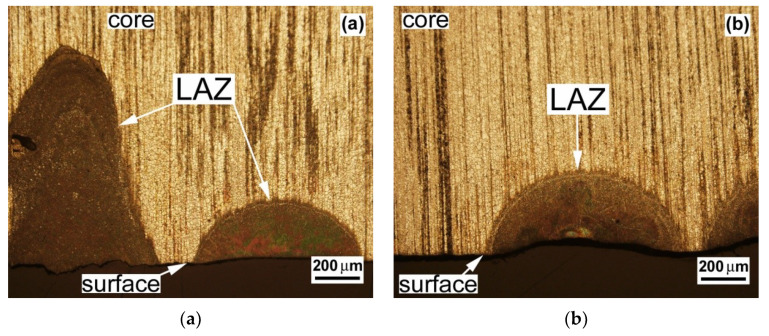
Light-optical micrographs showing the laser-affected zones (LAZs) created in AZ61 material after laser surface treatment at: (**a**) laser beam travel speed of 40 mm/s and laser beam defocusing of either +5 mm (left LAZ) or +10 mm (right LAZ); (**b**) laser travel speed of 20 mm/s and laser beam defocusing of +10 mm.

In the present investigation, the microstructural observations were focused on comparative studies of the morphological and microstructural characteristics of laser affected zones (LAZs), aimed at the optimization of the laser surface remelting conditions for obtaining optimal laser surface remelted microstructures without surface defects such as cracks, porosity, and ablation. Figure 2 shows typical cross-sectional LOM images of the LAZs created in the AZ61 base material by applying various laser surface remelting conditions.

Figure 2a shows the LAZs, i.e., the remelted layers formed after the laser treatments of the AZ61 alloy using the laser beam travel speed of 40 mm/s and various laser beam defocusings above the sample surface. It clearly indicates that whereas the use of a higher defocusing (i.e., +10 mm) of the laser beam produced a shallow, i.e., morphologically suitable, LAZ without material ablation (see the right portion of Figure 2a), the application of a lower defocusing (i.e., +5 mm) resulted in a significantly pronounced LAZ with a high depth of penetration and recognizable material ablation (see the left portion of Figure 2a). In order to also demonstrate the influence of laser beam travel speed, Figure 2b shows the LAZ created in the AZ61 material by the application of laser surface treatment using the already optimized laser beam defocusing (i.e., +10 mm) and a notably reduced laser beam travel speed of 20 mm/s. It can be clearly seen that the lowering of the beam travel speed resulted in significant material ablation, which represents rather undesirable surface degradation. Thus, it can be concluded that by using our 400 W laser power source, the optimal laser surface remelting conditions were found to be a 40 mm/s laser beam travel speed and a +10 mm laser beam defocusing above the sample surface. 

**Figure 3:**
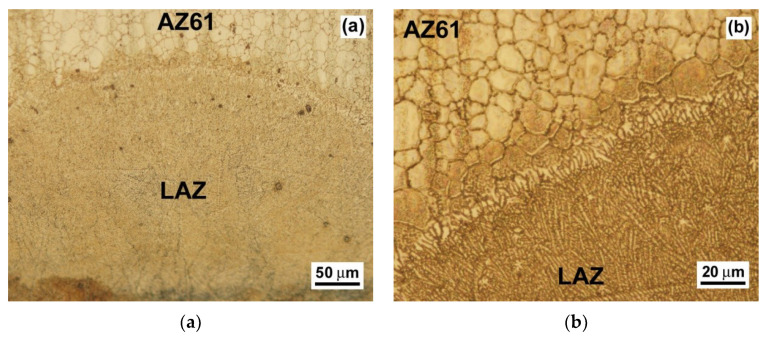
Detailed light-optical micrographs of the LAZ microstructure created using currently optimized laser surface treatment of AZ61 base material: (**a**) whole LAZ microstructural gradient and (**b**) interfacial region between the LAZ and unaffected AZ61 base material.

**Figure 4:**
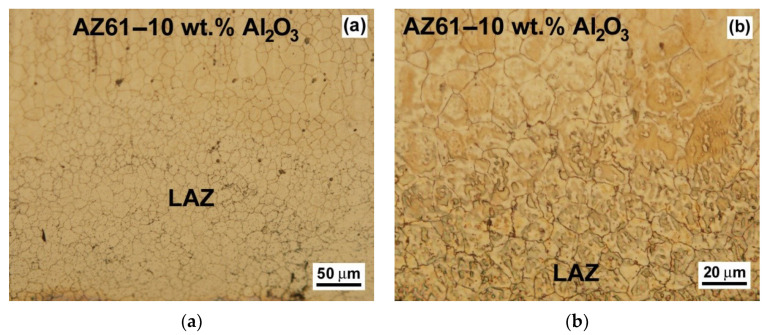
Detailed light-optical micrographs of the LAZ microstructure created using currently optimized laser surface treatment of AZ61–10 wt.% Al_2_O_3_ nanocomposite: (**a**) whole LAZ microstructural gradient and (**b**) interface between the LAZ and AZ61–10 wt.% Al_2_O_3_ composite.

The detailed LAZ microstructures created by the application of a currently optimized laser surface treatment are shown in Figure 3 and Figure 4. The presented micrographs illustrate possible growth morphologies that the solidifying metals may adopt. The LAZ in the AZ61 material is formed of a dendritic cell microstructure (Figure 3) which can be generally related to the local chemical instabilities at the solid–liquid interface during the alloy re-solidification as a result of the solute atoms’ migration into the liquid phase. The interfacial region, i.e., the fusion zone between the LAZ and the AZ61 base material did not show any cracks or other metallurgical defects (Figure 3). The influence of the optimized laser surface treatment on the LAZ characteristics of the AZ61–10 wt.% Al_2_O_3_ nanocomposite is shown in Figure 4.

It shows that the optimized laser treatment applied for the studied nanocomposite resulted in a crack-free, recrystallized-grain microstructure within the laser remelted zone (Figure 4). The performed microstructural analyses of the laser surface remelted samples of AZ61 alloy and AZ61–10 wt.% Al_2_O_3_ nanocomposite (Figure 3 and Figure 4) demonstrated significant microstructural differences between their individual LAZs. The thickness of the laser treated surface layer was about 250 μm (see Figure 3a) and 200 μm (see Figure 4a) for the AZ61 material and the AZ61-10 wt.% Al_2_O_3_ material, respectively. Regardless of the observed microstructural variations, it can be concluded that the use of currently optimized laser treatment conditions (i.e., 400 W, 40 mm/s, defocusing +10 mm) results in well-developed, defect-free LAZs without any pronounced ablation.

### 3.2. Nanoindentation Measurements

**Figure 5:**
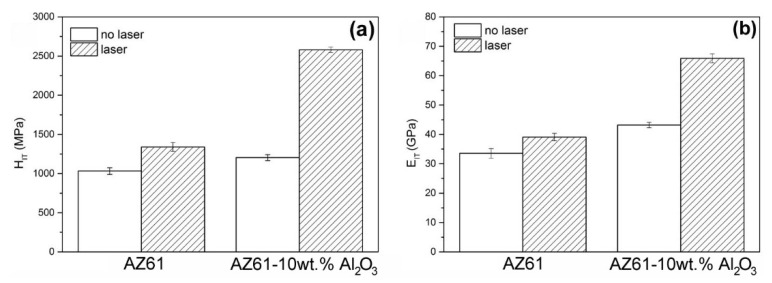
Nanoindentation characteristics of the investigated materials: (**a**) indentation nanohardness and (**b**) indentation modulus of elasticity.

In order to characterize local changes in the mechanical properties of the investigated materials after the application of optimized laser surface treatment, nanoindentation tests were carried out in both the laser unaffected as well as the laser affected areas. The nanoindentation properties of the laser treated materials were measured on metallographic cross-sections in central parts of the laser spots. Figure 5 shows graphical interpretations of the obtained nanoindentation characteristics, namely the indentation nanohardness (H_IT_) and the indentation modulus of elasticity (E_IT_) of the individual materials.

It can be clearly seen that both the application of laser surface remelting and the addition of Al_2_O_3_ nanoparticles results in clear improvements in the measured nanoindentation characteristics. Specifically, the nanohardness of the AZ61 base material increased by about 30% after the laser treatment. In the case of the AZ61–10 wt.% Al_2_O_3_ nanocomposite, the nanohardness increased more than twice (i.e., by about 114%) after the laser treatment. Furthermore, whereas the effect of laser surface remelting resulted in about a 17% increase in the elasticity modulus of the AZ61 base material, in the case of the AZ61–10 wt.% Al_2_O_3_ nanocomposite, it increased even by about 53%. The obtained results (Figure 5) indicate that the observed “dendrites-to-grains” microstructural modification (Figure 3 and Figure 4) plays a crucial role in improving the nanoindentation characteristics of the studied materials. This observation is in good agreement with similar findings of other studies about magnesium and aluminum alloys with incorporated nanoparticles [32,33,34,35]. Such laser-induced hardening behavior is expected to also enhance the tribological properties of the materials studied in the present work.

### 3.3. Friction and Wear Behavior

**Figure 6:**
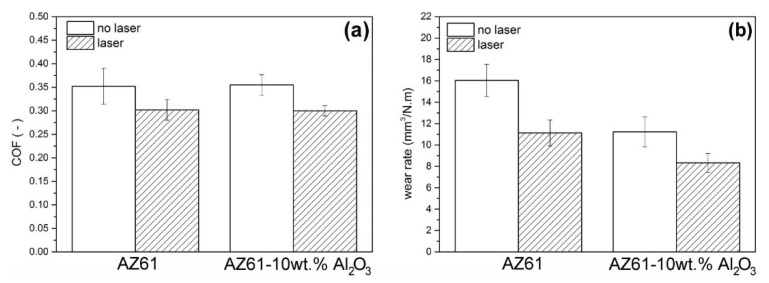
Tribological properties of investigated materials: (**a**) coefficient of friction and (**b**) specific wear rate.

From tribological tests of the investigated materials, the average values of the coefficient of friction (COF) and the specific wear rate were evaluated (see Figure 6).

Concerning the frictional behavior (Figure 6a), it can be stated that the addition of 10wt.% of Al_2_O_3_ nanoparticles into the AZ61 material resulted in only negligible changes in average COF. However, the application of laser surface treatment caused a clearly recognizable reduction in the average COF values of both the AZ61 material and the AZ61–10 wt.% Al_2_O_3_ nanocomposite. Regarding the wear rate (Figure 6b), the results show that the incorporation of Al_2_O_3_ nanoparticles within the AZ61–10 wt.% Al_2_O_3_ nanocomposite led to about a 30% decrease in its specific wear rate, compared to the AZ61 base material. Moreover, the application of laser surface remelting to the AZ61–10 wt.% Al_2_O_3_ nanocomposite, using optimized laser treatment conditions, led the wear rate to decrease by about 48%, compared to the laser untreated AZ61 base material. 

**Figure 7:**
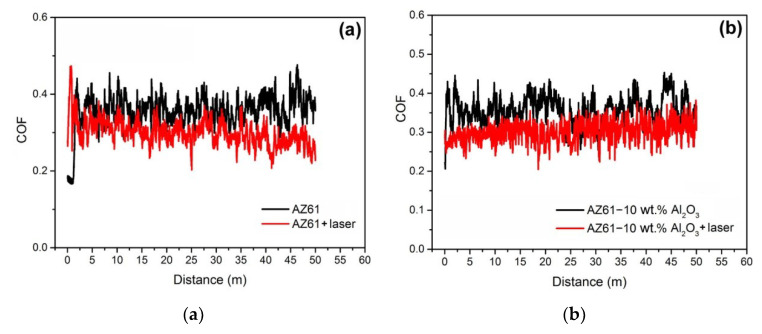
Variation of instantaneous COF values depending on the sliding distance during the tribological tests at a sliding speed of 100 mm/s and normal load of 5N for: (**a**) AZ61 base material; (**b**) AZ61–10 wt.% Al_2_O_3_ nanocomposite.

Figure 7 shows graphical interpretations of the instantaneous COF values depending on the sliding distance during the tribological tests. The presented graphical records indicate the continuous variation in the COF values for all the investigated materials and their material states with respect to the application of laser treatment. The observed variations are believed to be related to both the microstructural characteristics and the morphological evolution of the wear tracks’ surfaces during the tribological testing.

**Figure 8:**
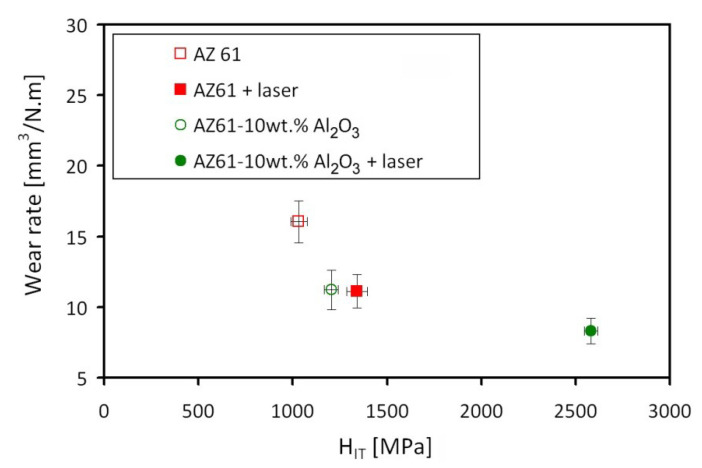
Correlation between indentation hardness and specific wear rate of studied materials.

Figure 8 shows the inverse proportionality between the material hardness and its specific wear rate. In other words, a higher hardness indicates a better wear resistance. 

The order of decreasing wear rate with increasing hardness is as follows: AZ61 (the highest wear rate at the lowest hardness), AZ61–10 wt.% Al_2_O_3_, AZ61+laser, AZ61–10 wt.% Al_2_O_3_+laser (the lowest wear rate at the highest hardness). The obtained results clearly show both effects, i.e., the effect of Al_2_O_3_ powder addition and the effect of laser surface remelting. The sole laser surface remelting of AZ61 base material showed quite a similar effect on the wear rate as the addition of Al_2_O_3_ powder into the AZ61 material without laser surface remelting. The best properties, i.e., the highest hardness and the lowest wear rate, were obtained by utilization of both effects, i.e., the Al_2_O_3_ powder alloying and the laser surface remelting. 

**Figure 9:**
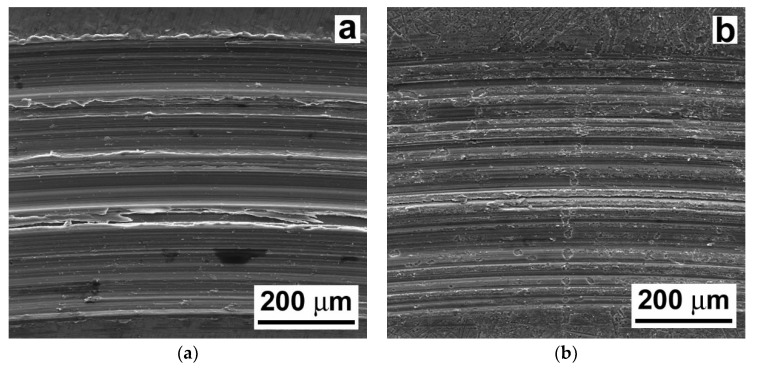
Scanning electron microscope (SEM) images showing the morphological characteristics of the wear tracks in the AZ61 base material: (**a**) the laser untreated and (**b**) the laser treated material state.

Figure 9 shows representative SEM images of the wear tracks created during the tribological testing of the AZ61 base material. The wear track surface morphology of the laser untreated AZ61 material is shown in Figure 9a and the wear track surface morphology of the laser treated AZ61 material is shown in Figure 9b. 

In both the material states, i.e., in the laser untreated as well as in the laser treated material state of the AZ61 base material, the dominant wear was abrasion and the governing wear micromechanism was microcutting, in addition to a small amount of plastic deformation and delamination. However, clear size differences in the individual morphological features were noticeable between the laser untreated and laser treated material states (Figure 9). Whereas deep and coarse abrasion grooves were typically present in the laser untreated AZ61 material (Figure 9a), fine and shallow abrasion grooves were typical for the laser treated AZ61 material (Figure 9b). 

**Figure 10:**
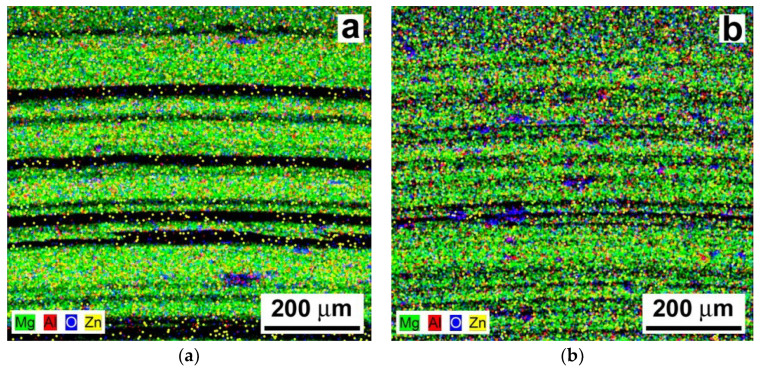
Energy dispersive X-ray (EDX) elemental maps from the wear track areas in AZ61 base material: (**a**) the laser untreated and (**b**) the laser treated material state.

Figure 10 shows the overall EDX elemental maps corresponding to the wear track areas depicted in Figure 9. Except for some localized occurrences of oxygen spots indicating tribologically induced surface oxidation, a quite homogeneous distribution of the individual chemical elements of the AZ61 material can be observed for both the laser untreated (Figure 10a) as well as the laser treated (Figure 10b) material state. However, it can be clearly seen that the wear track of the laser treated AZ61 material exhibited a higher fraction of oxidation, which was directly related not only to the heat of the tribological loading but also to the prior laser remelting process in the air environment.

**Figure 11:**
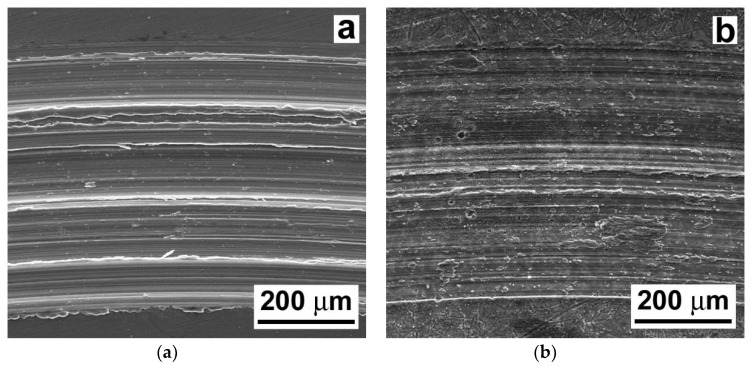
SEM images showing morphological characteristics of the wear tracks in AZ61–10 wt.% Al_2_O_3_ nanocomposite: (**a**) the laser untreated and (**b**) the laser treated material state.

Figure 11 shows representative SEM images of the wear tracks created during the tribological testing of the AZ61–10 wt.% Al_2_O_3_ nanocomposite. The wear track surface morphology of the laser untreated AZ61–10 wt.% Al_2_O_3_ nanocomposite is shown in Figure 11a and the wear track surface morphology of the laser treated nanocomposite is shown in Figure 11b. 

In contrast to the wear tracks of the AZ61 base material (Figure 9), the wear tracks of the AZ61–10 wt.% Al_2_O_3_ nanocomposite (Figure 11) indicate clear morphological changes related to the presence of Al_2_O_3_ nanoparticles. Especially in the case of the laser treated nanocomposite, the dominant wear types were both abrasion and adhesion (see Figure 11b). Apart from the clearly observed microcutting, the wear tracks of the AZ61–10 wt.% Al_2_O_3_ nanocomposite showed a higher amount of plastic deformation. Surface morphology has an important role in relation to the adhesiveness of the material. The SEM images in Figure 9 and Figure 11 revealed variant surface fragmentation and wear debris formation on the worn surfaces of the studied materials. Similar observations of wear debris formation within the wear tracks were also reported, e.g., in studies [36,37], which dealt with the wear properties of SiC-whisker and SiC-particulate/SiC-whisker reinforced aluminum composites. It can be assumed that in the present case, the formation of pronounced metal adhesive layers on the surfaces of the laser remelted materials (see Figure 9b and Figure 11b) might have acted as lubricants, reducing interfacial shear stresses during the tribological loading. Consequently, this resulted in a clear reduction in the corresponding COF values compared to those of the laser untreated materials (Figure 6). The obtained COF values are comparable, in principle, with those of the Mg-based composite materials [38]. 

**Figure 12:**
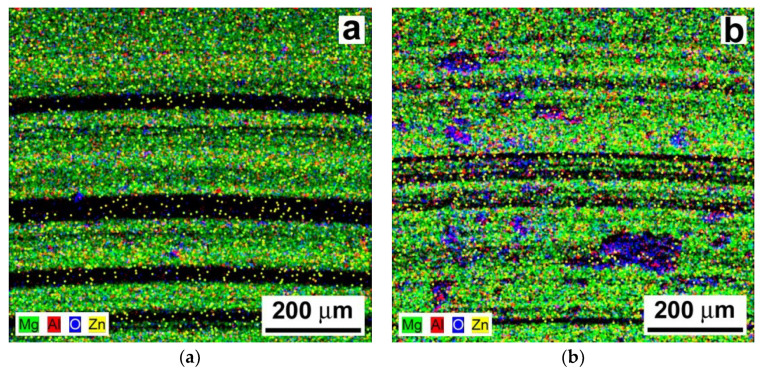
EDX elemental maps from the wear track areas in the AZ61–10 wt.% Al_2_O_3_ nanocomposite: (**a**) the laser untreated and (**b**) the laser treated material state.

Figure 12 shows overall EDX elemental maps corresponding to the wear track areas depicted in Figure 11.

Figure 12 indicates a quite homogeneous distribution of the individual chemical elements of the AZ61–10 wt.% Al_2_O_3_ nanocomposite for both the laser untreated and the laser treated material state. Similarly to the case of the tribologically tested AZ61 base material (Figure 10), the localized occurrence of oxygen spots along the wear tracks of the AZ61–10 wt.% Al_2_O_3_ nanocomposite indicate tribologically induced surface oxidation (Figure 12). The laser treated nanocomposite exhibited a higher amount of localized oxidation (Figure 12b) compared to the laser untreated composite (Figure 12a). The higher amount of localized oxidation in the laser treated composite (Figure 12b) is likely related to the laser treatment in the air environment, as well as being the effect of frictional heat during tribological loading. 

**Figure 13:**
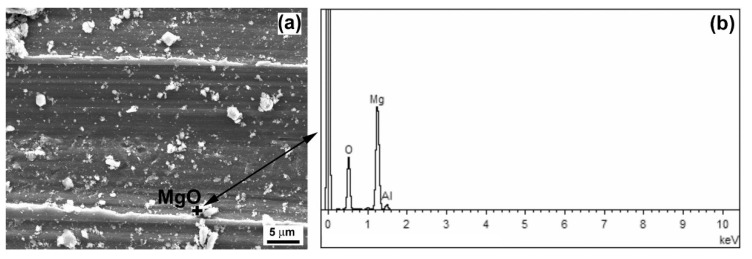
Detailed SEM analysis of the wear track of laser surface remelted AZ61–10 wt.% Al_2_O_3_ nanocomposite: (**a**) SEM image depicting the track morphology and (**b**) typical EDX spectrum of selected magnesium oxide.

Figure 13 shows a detailed view of the wear track on the laser remelted surface of the AZ61–10 wt.% Al_2_O_3_ nanocomposite with a typical EDX spectrum of magnesium oxide (MgO).

Summarily, it can be concluded that the tribological behavior of the studied magnesium alloy nanocomposites is mainly controlled by abrasion, adhesion, and surface oxidation processes.

## 4. Conclusions

In the present work, the ECAP-processed magnesium alloy AZ61 and its composite with 10 wt.% of Al_2_O_3_ nanoparticles were investigated in terms of the effect of laser surface remelting on their microstructure, hardness, and tribological behavior. Here are the main conclusions:

- The ECAP-processed AZ61 alloy and its derived AZ61–10 wt.% Al_2_O_3_ nanocomposite exhibited after the application of laser surface remelting significant microstructural differences within their laser-affected zones (LAZs). Whereas the LAZ microstructure of the AZ61 alloy had a typical dendritic character, the LAZ of the AZ61–10 wt.% Al_2_O_3_ nanocomposite showed a clearly recrystallized grain structure. The LAZs created by using optimized laser remelting conditions did not exhibit any serious metallurgical defects such as cracks, porosity, or ablation.
- The nanoindentation measurements revealed that the addition of Al_2_O_3_ nanoparticles into the AZ61 alloy and the application of laser surface remelting led to considerable improvements in their studied nanoindentation characteristics. Whereas the nanohardness of the AZ61 alloy increased by about 30% after the laser treatment, the nanohardness of the AZ61–10 wt.% Al_2_O_3_ nanocomposite increased more than twice after the laser surface remelting, compared to its laser untreated material state. The effect of laser surface treatment on the elasticity modulus was less pronounced. It increased by about 17% and 53% for the laser treated AZ61 alloy and the AZ61–10 wt.% Al_2_O_3_ nanocomposite, respectively.
- The results of the tribological tests indicated similar values for the average coefficient of friction (COF) for the ECAP-processed AZ61 alloy and the AZ61–10 wt.% Al_2_O_3_ nanocomposite, which were 0.352 and 0.355, respectively. The additional laser surface remelting led to a clear decrease in the average COF values for both the ECAP-processed AZ61 alloy and the AZ61–10 wt.% Al_2_O_3_ nanocomposite, which were 0.302 and 0.3, respectively. Thus, it can be concluded that, unlike the laser treatment, the addition of Al_2_O_3_ nanoparticles had quite an insignificant effect on the COF behavior in the individual material states under consideration.
- The observed effect of laser surface treatment on the lowering of the average COF values for both the ECAP-processed AZ61 alloy and the AZ61–10 wt.% Al_2_O_3_ nanocomposite was also acting synergically on the lowering of their specific wear rates. Whereas the incorporation of Al_2_O_3_ nanoparticles within the AZ61–10 wt.% Al_2_O_3_ nanocomposite led to about a 30% decrease in the specific wear rate compared to the AZ61 alloy, the additional laser surface remelting of the AZ61–10 wt.% Al_2_O_3_ nanocomposite led to a wear rate reduction of about 48%, compared to the laser untreated AZ61 base material.
- The microstructural variations related to the individual materials (i.e., AZ61 alloy and AZ61–10 wt. % Al_2_O_3_ nanocomposite) and their specific material states (i.e., either the laser untreated or laser treated) were clearly reflected by variations in their wear types and corresponding wear micromechanisms. Significant size differences in the individual morphological features (i.e., abrasion grooves, wear debris, plastically deformed areas) within the wear tracks were noticeable between the laser untreated and laser treated material states. Whereas the dominant wear type of the AZ61 alloy was abrasion and tribological laser remelting induced oxidation, the wear of the AZ61–10 wt.% Al_2_O_3_ nanocomposite included additionally occurring adhesion.

## Figures and Tables

**Table 1:** Chemical composition of AZ61 magnesium alloy as input material for preparation of AZ61–Al_2_O_3_ metal matrix composites.

Elements	Al	Mn	Zn	Si	Fe	Cu	Ni	Mg
(wt.%)	5.95	0.26	0.64	0.009	0.005	0.0008	0.0007	balance
